# Performance characteristics and quality assurance considerations for displays used in interventional radiology and cardiac catheterization facilities

**DOI:** 10.1002/acm2.12433

**Published:** 2018-08-09

**Authors:** Michael S. Silosky, Rebecca M. Marsh

**Affiliations:** ^1^ Radiology School of Medicine University of Colorado Aurora CO USA

**Keywords:** interventional radiology, image display, display luminance

## Abstract

**Purpose:**

While the performance of displays used for the acquisition and primary interpretation of medical images has been well‐characterized, notably absent are publications evaluating and discussing the performance of displays used in Interventional Radiology (IR) suites and Cardiac Catheterization (CC) laboratories. The purpose of this work was to evaluate the performance of these displays and to consider the challenges in implementation of display quality assurance practices in this environment.

**Methods:**

Ten large format displays used in IR and CC suites were evaluated. A visual inspection of available test patterns was performed followed by a quantitative evaluation of several performance characteristics including luminance ratio, luminance response function, and luminance uniformity. Additionally, the local ambient lighting conditions were evaluated.

**Results:**

Luminance ratios ranged from 243.0 to 1182.1 with a mean value of 500.1 ± 289.2. The maximum deviation between the luminance response function and the DICOM Grayscale Standard Display Function ranged from 11.2% to 38.3% with a mean value of 26.2% ± 10.9%. When evaluating luminance uniformity, the mean maximum luminance deviation was 13.2% ± 3.5%. The mean value of luminance deviation from the median was 7.8% ± 1.0%. Measured values of background illuminance ranged from 29.1 to 310.0 lux with a mean value of 107.6 lux ± 80.4 lux. While no mura or bad pixels were observed during visual inspection, damage including scrapes and scratches as well as smudges was common to most of the displays.

**Conclusion:**

This work provides much needed data for the characterization of the performance of the large format displays used in IR and CC laboratory suites. These data may be used as a point of comparison when implementing a display QA program.

## 1 INTRODUCTION

In recent years, there have been a number of publications characterizing the performance of displays used to present medical images.^1^, ^2^ Recommended performance criteria as well as guidelines and accreditation requirements for display quality assurance (QA) have been published for devices used for both primary interpretation of medical images (diagnostic displays)^3^ as well as those used as part of image acquisition systems (modality displays).^4^ Notably absent from the literature are publications evaluating and discussing the performance and QA of displays used in Interventional Radiology (IR) suites and Cardiac Catheterization (CC) laboratories. The purpose of this work was to fill this gap in knowledge.

The displays integrated with imaging systems in IR and CC facilities are used differently than those in other areas of diagnostic radiology. Images provided by these devices are used to guide procedures in real time rather than being used for primary interpretation. The IR and CC environment is also much different from a radiologist reading room — the lighting conditions are more variable, and the display is often mobile, leading to changing viewing conditions and the risk of collisions with other objects in the room. While these displays may be strictly classified as modality devices since they are directly attached to the acquisition system, their specific usage in guiding clinical care suggests that they should perform more similarly to diagnostic displays.

This study had two specific objectives. The first was to evaluate the performance of a cohort of displays used in IR and CC suites. Emphasis was placed on testing the displays in their native environment and under clinical operating conditions. The second objective was to consider the challenges in implementing a display quality assurance program within the practice environment of IR and CC facilities.

## 2 MATERIALS AND METHODS

It has become common practice for a single, large display to be used for both images and other clinical information when performing IR and CC procedures. These displays often receive input from a number of systems, and the user may determine where on the display to present the various forms of information. This includes acquired images, live fluoroscopy, and fluoroscopy acquisition parameters, as well as other information such as ultrasound images and patient vital signs. This study evaluated the large format displays used in ten IR and CC x‐ray angiography systems, each located in a separate suite within a single facility. It should be noted that, although some angiography systems use an array of smaller displays as opposed to a single large device, this type of configuration was not evaluated as part of this study.

Since the goal was to evaluate these devices as they are commonly used following installation by the manufacturer, no modifications were made to the grayscale lookup tables. Additionally, testing was performed with the displays set up in the same location used during clinical procedures. Evaluation of these devices began with a visual inspection of available test patterns followed by a quantitative evaluation of the following performance characteristics: diffuse reflectance, luminance ratio, luminance response function, and luminance uniformity. Additionally, the local ambient lighting conditions were evaluated.

All IR and CC displays with light meters that sense changes in ambient lighting conditions and modify display output are typically disabled at this facility. As such, the devices tested in this study were evaluated in this condition. It should be noted that two of the displays evaluated were installed with an additional protective screen made of a clear Plexiglas‐like material. Because the screens are in place during clinical use, all testing for these systems was performed under this condition.

The displays evaluated in this study included models made by Eizo (Eizo, Inc., Cypress, CA) and Fimi (Barco, Inc., Duluth, GA, USA) as part of x‐ray angiography systems made by Siemens (Siemens Medical Solutions, Malvern, PA, USA) and Philips (Philips Healthcare, Andover, MA, USA), respectively. Table 1 lists the display models and manufacturers for each device evaluated. Each display has been assigned a number, one through ten, that will be used to identify it for the remainder of this work. Given the relatively small sample size for individual display models, the results of this work should not be viewed as a comprehensive characterization for any particular display model.

**Table 1:** The assigned display number, display manufacturer, model number, size, and matrix for each display evaluated during this study are provided. Additionally, the manufacturer of the imaging system the display was installed with has been included

Number	Imaging system manufacturer	Display manufacturer	Model	Size (in)	Matrix
1	Siemens	Eizo	10293009	56	3840 × 2160
2	Siemens	Eizo	10656046	60	3840 × 2160
3	Siemens	Eizo	10656046	60	3840 × 2160
4	Siemens	Eizo	10656046	60	3840 × 2160
5	Siemens	Eizo	10656046	60	3840 × 2160
6	Siemens	Eizo	10656054	55	3840 × 2160
7	Philips	Fimi	CV56DS	56	3840 × 2160
8	Philips	Fimi	CV56DS	56	3840 × 2160
9	Philips	Fimi	CV56DS	56	3840 × 2160
10	Philips	Fimi	CV56DS	56	3840 × 2160

Also of note is that the arrangement of images within a large format display space can be customized, often resulting in several configurations used for any individual display. As such, images that take up half of the display for one operator may be much smaller and in another location of the display for a different operator. For facilities with a large number of suites and operators, it becomes quite difficult to evaluate every possible display configuration. Consequently, in this study, test patterns were displayed in the configuration with which the system was most recently used.

### 2.A Visual inspection

For each display, several test patterns were inspected visually (i.e., qualitatively). Uniform images were displayed at both minimum and maximum luminance and the displays evaluated for local nonuniformities including bright or dark mura, stuck pixels, and damage. Six of the displays in this study had a manufacturer‐loaded TG‐18 QC test pattern^5^ while the remaining four devices came loaded with the Society of Motion Picture and Television Engineers (SMPTE) test pattern.^6^ These patterns were inspected visually with emphasis placed on the visibility of the high‐contrast resolution patterns as well as the 0/5% and 95/100% contrast patches. Additionally, each display was evaluated for cleanliness before quantitative tests were performed. Displays that were found to be particularly “dirty” were cleaned until all foreign materials were removed from the surface.

### 2.B Diffuse reflectance

To evaluate the luminance characteristics of a display, one must first consider the effects of ambient illuminance and display reflectance. There are several ways in which ambient lighting can affect the perception of displayed images. Typically, these effects can be separated into two categories: those caused by specular reflection and those caused by diffuse reflection.^5^ Specular reflection tends to result in images of light sources or objects being superimposed on the displayed image. Because specular reflection is nonuniform and varies greatly with the ambient lighting conditions, it is best managed by avoiding displays with glossy front panels (which tend to be highly reflective) and encouraging the use of indirect lighting.

Unlike specular reflection, diffuse reflection does not result in clear images of individual light sources but as a general increase in the intensity of light leaving the surface of the display. Light from diffuse reflection is always present (unless the room is completely dark), but the impact can be minimized by appropriately adjusting a display's luminance response function. Consequently, any evaluation of a display's luminance response must consider the intensity of the light being reflected from the display. Ambient luminance (L_amb_) is the portion of the room illuminance that is reflected from the display toward the user and is expressed in units of cd/m^2^. It is directly dependent on the ambient lighting conditions and the reflective characteristics of the display and may be estimated as:

(1) $$L_{\mathrm{amb}}=E\;x\;R_{d}$$

where E is the ambient illumination incident on the display in lux, and R_d_ is the coefficient of diffuse reflection for the display. Therefore, R_d_ of a display can be calculated as the ratio of L_amb_ and E, both of which can be measured under controlled experimental conditions.^5^

In recent years, complex methodologies have been developed to characterize display reflectance in great detail. These include placing the display in a specially constructed reflector as a way to conduct measurements under hemispherical diffuse illumination conditions explicitly including the effects of specular reflection.^7^ Given the difficulty in conducting this type of measurement in a clinical setting, a more practical approach was used to estimate R_d._

**Figure 1:**
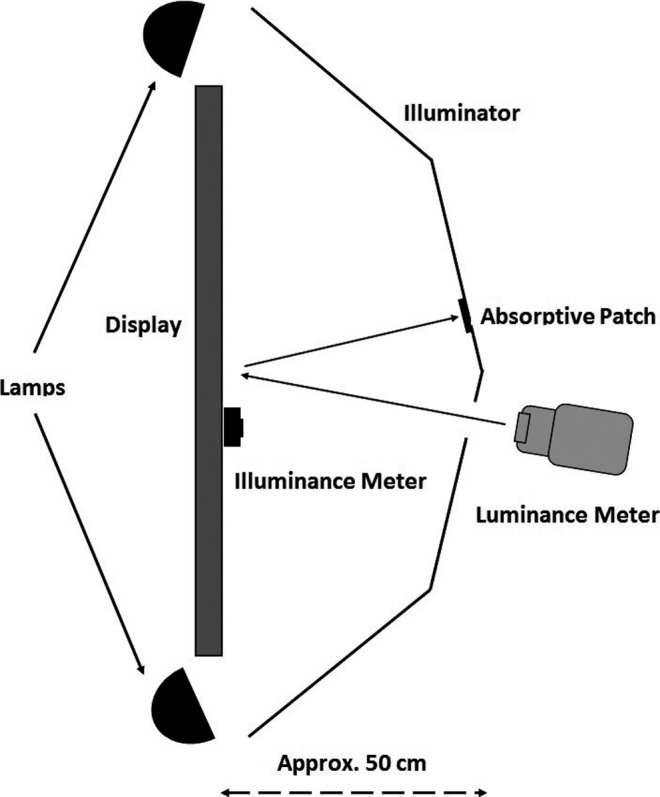
A schematic diagram of the experimental setup used to estimate the coefficient of diffuse reflection, R_d_. Care was taken to ensure that no other light sources were present and no direct line of sight lay between the lamps and the display surface. To ensure a consistent ambient illumination between tests, the distance between the illuminator and the display surface was maintained at 50 cm.

For each display listed in Table 1, R_d_ was estimated using the method described in Section 4.2.4.1.2 of AAPM Online Report No. 3, commonly referred to as TG‐18.^5^ The experimental setup used here mimicked the one shown in fig. 19(c) of the same report. Figure 1 illustrates the position of the light sources, illuminator, luminance and illuminance meters, and the absorptive patch used in this study.

For each display, L_amb_ and E were measured five times sequentially, and five independent values of R_d_ were calculated. L_amb_ was measured using a calibrated Konica‐Minolta CS‐100a Color Meter (Tokyo, Japan); E was measured using a calibrated RaySafe Unfors Xi light meter (Billdal, Sweden). The mean, standard deviation, and coefficient of variation were calculated for L_amb_ and E. Additionally, the mean value and the error in the mean were calculated from the five values of R_d_. It should be noted that the displays were often dirty and/or smeared with dried, unidentifiable fluids. These conditions tend to increase the amount of diffuse reflection. As such, a single display that had a large amount of visible residue from dried cleaning solution was tested twice, once before and once after cleaning. Additionally, some displays use in IR or CC suites have a removable external plastic cover used to protect the device. A single device of this type was evaluated as part of this study with reflectance tests being performed both with and without the cover in place.

### 2.C Background illuminance

Even for a single system, ambient lighting conditions in an IR or CC room may vary substantially depending on the type of procedure being performed and the preference of the performing physician. Since ambient illuminance is important when evaluating luminance response, it was necessary to get a sense of typical lighting conditions for the displays being evaluated. To investigate this, IR and CC staff and physicians were informally asked about how bright they typically leave the lights during a procedure. This information was used to adjust the lights in each suite, and the display was positioned as it would be during clinical use. The RaySafe Unfors Xi light meter was then used to measure the ambient illuminance. The mean value, standard deviation, and median were then calculated.

### 2.D Luminance ratio

The ability of a display to accurately present image data is primarily determined by its luminance ratio and luminance response function. Careful attention to these performance characteristics helps to maximize the amount of information that can be visualized on a display. Proper calibration helps the user perceive contrast in the image data and allows consistent image presentation across the imaging chain. The ACR‐AAPM‐SIIM Technical Standard for the Electronic Practice of Medical Imaging recommends LRs and Luminance Response functions for different types of displays.^3^

Luminance Ratio is calculated as:

(2) $$LR=\frac{\left(L_{\max}+L_{\mathrm{amb}}\right)}{\left(L_{\min}+L_{\mathrm{amb}}\right)}$$

where L_max_ and L_min_ are the maximum and minimum luminance, respectively, that the display is capable of producing. (L_amb_ is the ambient luminance, as defined in eq. (1)). All luminance measurements, except for L_amb_, were made using the RaySafe Unfors Xi light meter. (The method for determining L_amb_ is described in Section 2.B.) For each display, L_max_ and L_min_ were measured using white and black images, respectively, representing the maximum (white) and minimum (black) grayscale values for the display. L_amb_ was calculated for each display using eq. (1) and the previously determined values of R_d_ and ambient illuminance for each device. Equation (2) was then used to determine the luminance ratio of each display.

### 2.E Luminance response function

To evaluate the luminance response of a display, it is necessary to sample the luminance output across a range of grayscale values. A number of test patterns are available for this purpose. As previously stated, six displays in this study came from the manufacturer loaded with the TG‐18 QC test pattern while the remaining four devices came loaded with the SMPTE pattern. The luminance was measured for each luminance step available in the preloaded test pattern. The luminance response was then determined from these measurements. It should be noted that while the TG‐18 QC pattern consists of eighteen luminance steps, the SMPTE pattern only has eleven.

Since the RaySafe Unfors Xi light meter is a contact photometer, it was necessary to add the estimated L_amb_ (described in Section 2.B.) to each luminance measurement value. Next, the luminance values were converted to Just Noticeable Difference (JND) indices using the method described in TG‐18 Section 4.3.1.^5^ Additionally, the change in luminance per luminance value (dL/L) was calculated between each luminance step and plotted as a function of JND index for each display. Lastly, the luminance response of each display was compared with the DICOM Grayscale Standard Display Function (GSDF) by calculating the deviation between the measured values of dL/L and the ideal GSDF dL/L as a percentage of the ideal values. The maximum deviation was identified for each display.

### 2.F Luminance uniformity

To evaluate luminance uniformity, a white image was displayed on each device. Maximum luminance was chosen for two reasons. First, nonuniformities tend to be exaggerated at this level, representing a “worst‐case” scenario. Second, the authors wished to perform these measurements with images available on the system as it comes from the vendor, and some systems did not come with traditional uniformity test images installed. Additionally, these devices are seldom used to display a single image covering the entire active surface. Consequently, uniformity was evaluated over the largest portion of the device used for image display. This was typically the section of the display that was used to show live fluoroscopy. As previously mentioned, these devices may be configured a number of ways and QA testing should be designed to consider the specific usage of each system. Luminance was measured in nine locations across the active portion of the display including the center, near the corners, and near the center of each edge. Two uniformity metrics, the maximum luminance deviation (MLD), and the maximum luminance uniformity deviation from the median (LUDM), were calculated for each display. MLD is calculated as:

(3) $$MLD=\frac{Max-Min}{Max+Min}\;\times 200$$

where Max and Min are the maximum and minimum measured luminance, respectively. LUDM is calculated as:

(4) $$LUDM=Maximum\;\left(\frac{\left|N-Med\right|}{\mathrm{Med}}\;\times 100\right)$$

where N is the measured luminance at each of the nine locations and Med is the median value of those measurements. Additionally, the mean value, standard deviation, and coefficient of variation were calculated across all displays.

## 3 RESULTS

### 3.A Visual inspection

**Figure 2:**
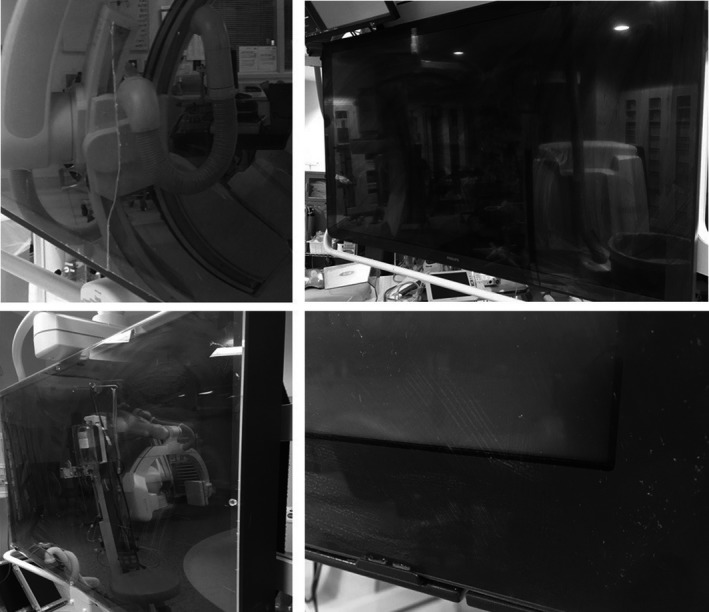
A photograph of a large crack running vertically through the protective cover used with a large format display (upper left). Streaking often occurs when sterile cleaning of these devices leaves a large amount of residue (upper right and lower left). Additionally, drops of iodinated contrast material left on the screen may dry to provide a sticky residue that is difficult to remove (lower right).

The resolution patterns and contrast patches were visible for every display evaluated in this study. While no mura or bad pixels were observed on any of the displays evaluated in this study, the visual inspection revealed some items of interest. First, every display had varying amounts of dried contrast media or other unidentifiable fluids speckled onto the surface of the display. Second, recent cleaning by clinical staff was often incomplete, leaving contrast and other fluids remaining on the display and creating streaks left by the cleaning agents. Third, a large, vertical crack was present in the protective cover of one display. Figure 2 provides photographs of the some of these observations.

### 3.B Diffuse reflectance

The mean values of R_d_ for individual displays ranged from 0.0013 cd/m^2^ per lux to 0.0077 cd/m^2^ per lux with a mean across all displays of 0.0038 ± 0.0025 cd/m^2^ per lux. The median value across all displays was 0.0032 cd/m^2^ per lux. Table 2 lists the mean value, standard deviation, and coefficient of variation for E and L_amb_, as well as the mean value, error in the mean, and percent error for R_d_.

**Table 2:** The mean values, standard deviation, and coefficient of variation for both E and L_amb_ as well as the mean and error as a percentage for R_d_ have been listed. Due to the relatively low error in measurements of Illuminance, error in R_d_ tends to be dominated by variation in measurements made by the telescopic photometer**.**

Display	E (lux)			L_amb_ (cd/m^2^)			$R_{d}\;\left(\frac{\frac{\mathrm{cd}}{m^{2}}}{\mathrm{lux}}\right)$	
	Mean	St. Dev.	COV (%)	Mean	St. Dev.	COV (%)	Mean	%
1	177.05	0.07	0.04	0.52	0.04	6.87	0.0029	6.87
2	173.14	0.17	0.10	0.27	0.005	2.00	0.0016	2.00
3	160.5	0.22	0.14	0.23	0.009	3.82	0.0015	3.82
4	166.88	0.13	0.08	0.22	0.01	6.43	0.0013	6.43
5	186.86	0.24	0.13	0.67	0.01	1.99	0.0036	1.99
6	187.06	0.43	0.23	0.70	0.02	2.76	0.0037	2.77
7	170.2	0.71	0.42	1.20	0.04	3.19	0.0071	3.22
8	188.3	0.34	0.18	1.45	0.01	1.02	0.0077	1.04
9	185.3	0.25	0.14	0.26	0.01	3.24	0.0014	3.25
10	191.32	0.44	0.23	1.27	0.02	1.63	0.0067	1.64

Initially, Display 2 had particularly severe streaking from dried cleaning agents. This display was tested both before and after remedial cleaning. Before cleaning, the mean value of R_d_ was 0.0055 cd/m^2^ per lux with an error in the mean of 0.0001 cd/m^2^ per lux, as opposed to the mean R_d_ value after cleaning of 0.0016 cd/m^2^ per lux listed in Table 2.

### 3.C Background illuminance

Measured values of background illuminance ranged from 29.1 to 310.0 lux with a mean value of 107.6 ± 80.4 lux. The median value was 101.8 lux. Individual measurements for each display are listed in Table 3.

**Table 3:** The measured values of L_min_, L_max_, and room illuminance, as well as calculated values of R_d_, L_amb_, and LR for all 10 displays are provided. Additionally, the mean value, standard deviation, and median were calculated for each characteristic

1	0.66	280.5	0.0029	116.58	0.20	281.1
2	0.10	284.6	0.0016	73.79	0.14	1313.4
3	0.12	282.4	0.0015	119.86	0.33	958.7
4	0.90	333.3	0.0013	119.86	0.16	315.2
5	0.68	296.2	0.0036	150.00	0.54	243.0
6	0.39	362.0	0.0037	134.20	0.50	407.0
7	0.59	296.5	0.0071	87.00	0.61	246.7
8	0.34	339.4	0.0077	42.65	0.33	507.9
9	0.38	327.6	0.0014	42.88	0.06	745.2
10	0.51	284.2	0.0067	29.10	0.19	404.1
Mean	0.47	308.7	0.0037	91.59	0.31	542.2
St. Dev.	0.25	29.3	0.0025	42.77	0.19	356.6
Median	0.45	296.4	0.0033	101.79	0.26	405.5

### 3.D Luminance ratio

Calculated values of LR ranged from 243.0 to 1182.1 with a mean value of 500.1 ± 289.2. The median value was 405.5. Individual values for each display are listed in Table 3.

### 3.E Luminance response function

**Figure 3:**
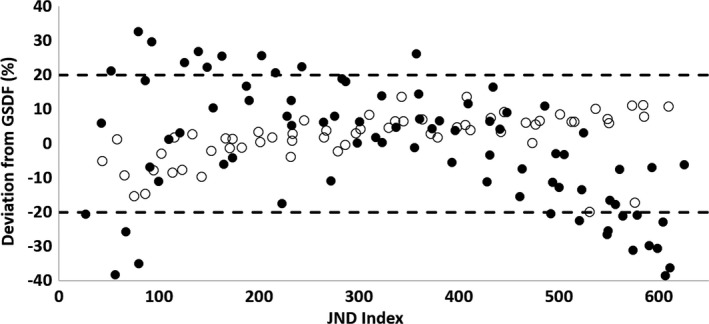
The deviation from the DICOM GSDF as a percentage is plotted vs the Just Noticeable Difference Index for each luminance step measured on each device. Solid circles represent measurements from displays that exceeded 20% deviation of at least one luminance level. Open circles represent measurements made on devices that never exceeded 20% deviation from the GSDF function. The dashed lines indicate a deviation of ±20%. These deviations were observed to be most severe for luminance measurements made near the extremes of the display function, i.e., minimum and maximum luminance.

The maximum deviation from the DICOM GSDF ranged from 11.2% to 38.5% with a mean value of 26.2% ± 10.9%. Table 4 lists the maximum deviation and mean deviation from the DICOM GSDF for each display. It should be noted that the luminance response function of six of the ten displays deviated from the GSDF by greater than 20% for at least one grayscale step. Figure 3 plots the luminance response deviation from the GSDF (Eq. (3)) vs JND Index for each luminance step measured on each display. GSDF compliant displays are shown with an open circle while noncompliant displays are shown with a solid circle. For these displays, the most extreme deviations were typically near minimum and maximum luminance.

**Table 4:** The maximum and mean deviation from the DICOM GSDF is listed for each display. The mean value across all displays as well as the standard deviation and coefficient of variation has been calculated for both metrics

Display	Max deviation (%)	Mean deviation (%)
1	11.2	5.8
2	31	17.7
3	15.4	5.1
4	14.7	6.5
5	35	16.6
6	25.7	8.5
7	32.7	14.6
8	38.3	22.1
9	38.5	9.7
10	19.9	7.3
Mean	26.2	11.7
St. Dev.	10.9	5.9
COV	0.4	0.5

It should be noted that two distinct behaviors are apparent. These data suggest that some displays were calibrated to the DICOM GSDF while others were calibrated with a linear lookup table.

### 3.F Luminance uniformity

The mean value of MLD was 13.2% ± 3.5%. The mean value of LUDM was 7.8% ± 1.0%. These values correspond to coefficients of variation of 26.2% and 13.2%, respectively. Table 5 provides MLD and LUDM for each device.

**Table 5:** The calculated value of MLD and LUDM is provided for each display tested as well as the mean value, standard deviation, and coefficient of variation across all displays

Display	MLD (%)	LUDM (%)
1	15.9	8.0
2	17.6	8.9
3	17.2	8.8
4	14.1	8.0
5	14.2	7.5
6	13.6	8.4
7	11.4	8.0
8	8.3	6.1
9	11.8	8.2
10	7.4	5.9
Mean	13.2	7.8
St. Dev.	3.5	1.0
COV	26.2	13.2

## 4 DISCUSSION

### 4.A Visual inspection and local nonuniformities

As mentioned, resolution and low‐contrast patches were clearly visible for all displays tested, regardless of other performance metrics. Given that many of these displays were not DICOM GSDF compliant, this raises questions regarding the utility of visual inspection of these objects. While an inspection of the 5% contrast patches at the low and high end of the luminance scale may identify extremely underperforming displays, it is unlikely to provide a meaningful visual analysis. It should be noted that the TG‐18 QC test pattern has other contrast objects such as the low‐contrast corner boxes and QUALITY CONTROL objects. An inspection of these test objects may provide greater utility when evaluating displays. However, a test pattern with a wider range of contrast variations over the entire luminance range, such as the TG‐18 PQC test pattern, may be more useful for this purpose.

No local nonuniformities resulting from poor display function (i.e., stuck pixels or bright or dark mura) were observed. However, the display surface created nonuniformities in several displays. As mentioned, cracks, scratches, and smudges, some of which were large, were common. Therefore, implementing a proper cleaning regimen and evaluating displays for physical damage seems a necessary part of a quality assurance program. While the devices tested in this study received routine cleaning from clinical staff, the sterilizing wipes used for this purpose typically contain active quaternary ammonium chlorides that tend to leave a residue on clear plastics.^8^ In extreme cases, this residue can interfere with visualization or substantially increase reflectance as reported in Section 3.B. Also, dried fluids, including blood but particularly contrast agents, tended to not be fully removed by the initial cleaning by clinical staff. Consequently, in addition to an initial cleaning for infection control purposes, it may be useful to follow with a cleaning solution designed specifically to reduce residue while preventing damage to the display surface.

### 4.B Measurement of display reflectance

As stated in Section 3.B., the mean value for R_d_ was 0.0038 cd/m^2^ per lux. This value is slightly lower than for a diagnostic display, which typically has an R_d_ between 0.005 and 0.010 cd/m^2^ per lux.^9^ Given that IR and CC suites may have significantly brighter lighting than a typical reading room, the displays used with these systems may have been designed to minimize the reflective properties. However, in interpreting these results, it is important to consider limitations of the technique described by TG‐18 for measuring R_d_. The TG‐18 methodology eliminates discrete light sources and minimizes the effect of specular reflection by using a dark absorptive patch (Fig. 1). However, by removing the contribution of specular reflections from diffuse light sources, values of R_d_ determined in this fashion may underestimate the amount of light being reflected toward a viewer.

Given the challenge in measuring reflectance, it may be reasonable for some facilities to assume standard values of R_d_ when evaluating displays. It is relatively simple to measure illumination for individual displays, making it straightforward to determine L_amb_. Ambient light can then be accounted for when evaluating luminance response. One possible method would be to assume a value of 0.004 cd/m^2^ per lux for all displays of this type (i.e., large format displays used as part of an IR or CC angiography system), based on the observations of this study. However, it should be noted that this work evaluated a relatively small number of display models and that this single value may not be appropriate for other models. Additional work should be done to confirm or refute the validity of this assumption.

Repeat measurements demonstrate good consistency, with the error in the mean of R_d_ ranging from 1.04% to 6.87%. As shown in Table 2, the error in the illuminance measurement was very small, indicating that the error in R_d_ was dominated by variations in the telescopic photometer measurements. It should be noted that displays 5 and 9 had a value of R_d_ that varied substantially from other displays of the same model type while still falling within the typical range across all displays tested. During evaluation, it was observed that the thickness of the front glass appeared to vary among devices of the same model. It is unclear if this is the reason for inconsistency in values of R_d_ and further investigation is necessary.

### 4.C Luminance ratio and luminance response

According to the ACR‐AAPM‐SIIM Technical Standard for Electronic Practice of Medical Imaging, medical displays used for diagnostic interpretation should have a luminance ratio of at least 350. Other displays should have a luminance ratio of at least 250.^3^ As shown in Table 3, eight of the ten displays had a LR above 250; six of these eight displays exceeded a LR of 350. Two displays had an LR between 240 and 250. These observations suggest that the LR of large format displays used in IR and CC suites is often at least as high as the recommendations put forth in the technical standard for nondiagnostic displays. Consequently, a QA program that requires that the LR is at or around 250 may be appropriate.

The relationship between L_amb_ and L_min_ is often overlooked. According to TG‐18, the value of L_min_ should be at least four times L_amb_. This is necessary to prevent fluctuations in room lighting from negatively affecting contrast at low luminance levels.^5^ As shown in Table 2, only a handful of displays met this criterion with the mean value of L_min_ only 1.5 times the mean value of L_amb_. Generally, increasing the value for L_min_ on these devices (while ensuring that L_max_ is high enough to obtain an adequate luminance ratio) would help avoid loss of contrast at low gray levels.

**Figure 4:**
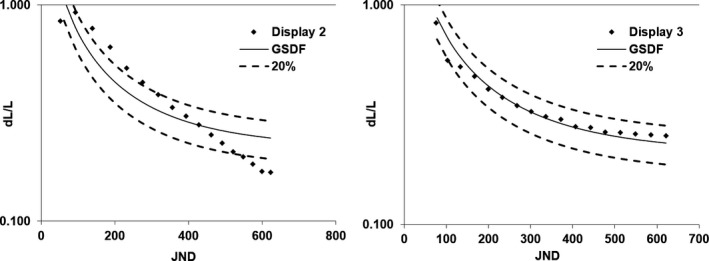
The change in luminance per luminance level vs just noticeable difference index is plotted for displays 2 and 3 along with the ideal performance as determined by the DICOM Gray Scale Display Function and the ±20% criterion. These displays have the same model number and were installed within a year of each other. Luminance response is observed to vary greatly between these two devices with display one falling outside the 20% criterion provided by the ACR‐AAPM‐SIIM Technical Standard for Electronic Practice of Medical Imaging.

Unlike luminance ratio, the luminance response for these displays was not consistent with criteria put forth in the technical standard, which recommends that for diagnostic displays, the luminance response is within 10% of the DICOM GSDF. A deviation of 20% is recommended for all other display types.^3^ As stated in Section 3.E., only four of the ten displays tested met the 20% criterion at all tested luminance levels. Interestingly, the four displays that were within 20% were different models. In fact, there was markedly different behavior in two displays of the same model, installed on the same type of imaging system, and manufactured within a year of each other (Displays 2 and 3). This difference can be appreciated by looking at the luminance response functions of each of these displays, plotted in Fig. 4. as the change in luminance per luminance level (dL/L) vs JND. The ideal performance function, based on the DICOM GSDF and the ±20% criterion, is also shown.

Figures 3 and 4 suggest that some displays have been calibrated to the DICOM GSDF while others use a linear lookup table. They also suggest that upon acceptance testing of IR and CC suites, the GSDF compliance of the display should be evaluated to ensure consistent image appearance across all devices. In the case where large deviations are seen from the DICOM GSDF, one should consider having the vendor recalibrate the display (i.e., adjust the lookup table).

The evaluation of luminance ratio and luminance response raises an important question regarding what level of performance can and should be expected of these displays. While it is tempting to treat these devices similarly to other nondiagnostic displays, they are the primary means by which image information acquired during IR and CC procedures is conveyed to the performing physicians. Consequently, the quality of images on these displays has a direct impact on directing patient care. As such, the radiology community should consider treating these devices as similar to diagnostic displays and expect similar performance. Regarding luminance ratio, this seems to be achievable, with many of the devices in this study already performing at this level. However, given that the ambient lighting conditions are more variable than in a dedicated reading room and may change depending on the particular physician performing a case, the type of case being performed, and the position of the display during use, there may be some justification for maintaining the looser 20% criterion for luminance response.

### 4.D Quantitative luminance uniformity

As stated in Section 3.F., the mean values for MLD and LUDM were 13.2% and 7.8%, respectively. In discussing the quantitative evaluation of luminance uniformity, TG‐18 recommends that MLD, measured using the TG18 UN10 and UN80 test patterns, should be less than 30%. However, it is worth noting that these guidelines were focused on the performance of cathode ray tube displays. We are unaware of guidelines specific to the uniformity of displays used in IR and CC suites. While one could consider adapting the requirements for modality displays from the ACR 2012 Computed Tomography Quality Control Manual or the ACR 2015 Magnetic Resonance Imaging Quality Control Manual,^10^, ^11^ it is worth considering whether quantitative evaluations of display uniformity are useful as a quality assurance metric. Arguably, the human visual system tends to be more sensitive to the local nonuniformities identified in a visual inspection than global changes in uniformity across the face of the display. This approach is supported by the ACR‐AAPM‐SIIM Technical Standard for Electronic Practice of Medical Imaging, which does not provide a recommendation for quantitative luminance uniformity.^3^

Regarding LUDM, it has been suggested that a uniformity metric that compares measured values against the median might be a more appropriate metric than one that looks at the difference between minimum and maximum values.^12^ As one might expect, the values for LUDM were substantially lower than MLD for all displays. Additionally, the coefficient of variation across all displays was 13.2% for LUDM as opposed to the 26.2% observed for MLD. This suggests that LUDM is more consistent in the presence of outliers than MLD.

### 4.E Display QA and implementation challenges

The development and implementation of new quality assurance procedures can be challenging in any clinical environment. This is true for display devices as much as for the imaging modalities themselves. Often, imaging physicists have limited access to IR and CC suites and may only use the equipment during a scheduled annual test or after a major repair. Consequently, any QA procedures performed more than once a year may need to be conducted by personnel other than the physicist. While it may be justified to perform the quantitative evaluations annually, the prevalence of display uncleanliness, scratches, and other damage indicate that a visual inspection should be performed more routinely. A quick visual inspection of a limited number of test patterns, by personnel who have received adequate training from a physicist, can be performed in a matter of minutes. This will also provide an opportunity to ensure that display devices are properly cleaned.

Concerning quantitative measurements, an illuminance meter is necessary to evaluate the lighting conditions of the room and either a contact or telescopic photometer will be necessary to evaluate luminance response and uniformity. While it is outside the scope of this work to compare and contrast these devices, it should be noted that a telescopic photometer is necessary if the display reflectance methodology used in this work is to be implemented.

### 4.F Study limitations

This work serves to provide a much needed point of comparison regarding typical performance of displays used in IR or CC suites. However, there are a number of limitations that should be considered when interpreting the results. The most significant limitation to this study is that only ten displays, split between two separate vendors, were evaluated. Of these ten, four separate models were included. As such, it is difficult to draw conclusions about the performance of any specific model.

The remaining limitations are primarily related to experimental design. Luminance response was evaluated assuming ambient lighting conditions specific to the usage at a single institution. As stated, these conditions may vary based on the procedure and individual user preference. However, the results presented here may be used to identify typical performance characteristics for this class of displays, and the values for ambient light and reflectance may be useful when facility‐specific values are unavailable. Also, the methodology used to evaluate display reflectance relies on the elimination of discrete light sources, but this is prohibitively difficult in a clinical setting. While it can be generally assumed that physicians will position the display such that major specular reflections will not interfere with their ability to view images, these effects cannot be discounted entirely.

Another limitation is that the visual inspection of display uniformity was performed at only two luminance levels. It is possible that visible defects may be more apparent at other levels and an assessment performed at additional steps may be justified. All displays were evaluated with the test pattern in the portion of the display most commonly used for that system. This meant that for different displays, the test patterns were shown in different regions of the display. This approach does not encompass all possible display configurations, and the results from the current study may underestimate the MLD and LUDM. While the absolute luminance values may change across the display, it is unlikely that the deviation from the ideal GSDF function would change substantially.

Finally, an evaluation of display performance relative to viewing angle was not performed. The environment in which these displays are used may require the operator to view images at a variety of angles and luminance output may vary substantially. Display reflectance may also vary with viewing angle. Future work should include evaluation of these performance characteristics.

## 5 CONCLUSION

This work evaluated performance characteristics for large format displays used in IR and CC suites. The paucity of published data regarding these displays makes it difficult to compare our results to previous work. However, these data may be used as a point of comparison for other facilities and encourage the implementation of future display QA programs.

## CONFLICT OF INTEREST

The authors have no conflicts of interest.
